# Effects of an Optimized Aged Garlic Extract on Cardiovascular Disease Risk Factors in Moderate Hypercholesterolemic Subjects: A Randomized, Crossover, Double-Blind, Sustainedand Controlled Study

**DOI:** 10.3390/nu14030405

**Published:** 2022-01-18

**Authors:** Rosa M. Valls, Judit Companys, Lorena Calderón-Pérez, Patricia Salamanca, Laura Pla-Pagà, Berner Andrée Sandoval-Ramírez, Antonio Bueno, Jose Puzo, Anna Crescenti, Josep M. del Bas, Antoni Caimari, Aurora Salamanca, Alberto E. Espinel, Anna Pedret, Lluís Arola, Rosa Solà

**Affiliations:** 1 Functional Nutrition, Oxidation and Cardiovascular Diseases Group (NFOC-Salut), Facultat de Medicina i Ciències de la Salut, Universitat Rovira i Virgili, C/Sant Llorenç, 21, 43201 Reus, Spain; rosamaria.valls@urv.cat (R.M.V.); patricia.salamanca@urv.cat (P.S.); bernerandree.sandoval@urv.cat (B.A.S.-R.); rosa.sola@urv.cat (R.S.); 2 Eurecat, Centre Tecnològic de Catalunya, Unitat de Nutrició i Salut, Av/de la Universitat, 1, 43204 Reus, Spain; judit.companys@eurecat.org (J.C.); Lorena.calderon@eurecat.org (L.C.-P.); laurapla.dn@gmail.com (L.P.-P.); josep.delbas@eurecat.org (J.M.d.B.); lluis.arola@urv.cat (L.A.); 3 Lipid Unit, Clinical Analysis and Biochemistry Service, Hospital San Jorge de Huesca, 22004 Huesca, Spain; abueno27@hotmail.com (A.B.); josepuzo@gmail.com (J.P.); 4 Eurecat, Centre Tecnològic de Catalunya, Biotechnology Area, Av/de la Universitat, 1, 43204 Reus, Spain; antoni.caimari@eurecat.org; 5 Pharmactive Biotech Products, S.L., C/Faraday 7, 28049 Madrid, Spain; aespinel@pharmactive.eu (A.S.); aurora.salamanca.molina@gmail.com (A.E.E.); 6 Nutrigenomics Research Group, Department of Biochemistry and Biotechnology, Campus Sescelades, Universitat Rovira i Virgili, 43007 Tarragona, Spain; 7 Internal Medicine Service, Hospital Universitari Sant Joan de Reus, Av/del Doctor Josep Laporte, 2, 43204 Reus, Spain

**Keywords:** aged garlic extract, S-allyl-L-cysteine, cardiovascular disease risk factor, hypercholesterolemia, blood pressure, pulse pressure

## Abstract

The consumption of aged black garlic (ABG) has been related to improvements in several cardiovascular disease (CVD) risk factors. However, the extent of the beneficial effects depends on the garlic aging process and the amount and type of chemical compounds accumulated. The main objective of this study was to assess the effect of daily intake of a well-characterized ABG extract with a standardized S-allyl-L-cysteine (SAC) yield in combination with dietary recommendations regarding CVD risk factors in individuals with moderate hypercholesterolemia. Sixty-seven hypercholesterolemic individuals with low-density lipoprotein cholesterol levels ≥115 mg/dL were randomized in a crossover, double-blind, sustained, and controlled intervention study. The participants consumed 250 mg (1.25 mg SAC)/tablet/day ABG or a placebo for 6 weeks, with 3 weeks of washout. Blood and pulse pressure and other CVD risk biomarkers were determined at the beginning and end of each intervention. At 6 weeks, ABG extract reduced diastolic blood pressure (DBP) (mean (95% CI) −5.85 (−10.5; −1.3) mm Hg) compared to the placebo, particularly in men with a DBP > 75 mm Hg. The consumption of an improved ABG extract with 1.25 mg of SAC decreased DBP, particularly in men with moderate hypercholesterolemia. The potential beneficial effects of ABG may contribute to obtaining an optimal DBP.

## 1. Introduction

One of the most characteristic components of the Mediterranean diet is garlic (*Allium sativum* L.), which has been found to have protective properties against cardiovascular disease (CVD) risk factors, among other health effects, as recognized by international authorities [1,2].

Since ancient times, aged garlic has been produced with milder organoleptic characteristics than commonly consumed fresh garlic and interesting beneficial health effects. The production of aged black garlic (ABG) for health applications consists of a treatment (sometimes called “fermentation”) of the fresh garlic bulb at high temperature and controlled humidity for a period of weeks to several months. Garlic cloves become dark with a jelly-like consistency, and this process yields the appreciated culinary characteristics: a sweet taste without the pungent off-flavor of fresh crushed garlic. The aged bulb undergoes substantial biochemical changes that distinguish it from fresh garlic. The bulb tends to lose water and releases free sugars (especially fructose), which combine with amino acids and appear to be key intermediate compounds of the Maillard reaction [3]—furfurals [4] and melanoidins [5]—when browning. Soluble polyphenols and flavonoids also usually increase [6]. However, the most important change is related to organosulfur chemicals. Garlic, as observed in other Allium species, fixes inorganic sulfate (SO42–) as L-cysteine [7] and produces various organosulfur compounds with functional activity. Aging modifies the profile and quantities of those molecules. The main organosulfur compounds in fresh garlic (i.e., alliin and allicin) are downregulated by inactivation of alliinase and thermal degradation of the products [8,9,10]. Polysulfides, such as diallyl sulfide, diallyl disulfide, diallyl trisulfide, dithiins, and ajoene, appear after decomposition of allicin [11]. S-allyl-L-cysteine (SAC), which is virtually absent in fresh garlic, is synthetized and initially upregulated by deglutamylation of γ-glutamyl-S-allyl-cysteine and reaches several times higher concentrations than those in fresh tissues before its progressive catabolism to other compounds [12].

Industrial black garlic extracts have been customarily standardized with respect to only one of the major active compounds: SAC; however, no standardized aging process has been established. Very often, extracts are produced from aged bulbs transformed by the same simple traditional heat/humidity process [10,13], where temperature, humidity, and fermentation time vary depending on the manufacturer’s preferences.

Other kinds of extracts are produced from processed garlic, e.g., garlics submitted to bacterial fermentation. Other commercially successful extracts are produced from aged bulbs soaked in water or ethanol (simply called “aged garlic”), which contain accumulated SAC, but aged garlics show different chemical compositions of furfurals and organosulfur compounds than ABG extracts [14,15].

Pure SAC and processed garlic (aged and different aged black garlics) have shown healthy effects at multiple targets. SAC and ABG extracts show cardiovascular, antimicrobial, immunomodulatory, hepatoprotective, digestive system protective, antidiabetic, anti-obesity, neuroprotective, and renal protective properties in in vitro and in vivo tests [16,17].

However, in humans, evidence relying on clinical trials performed with different kinds of aged garlic (but not ABG) has been recently reviewed. Most human studies have been carried out with aged garlic and conducted in diagnosed patients with some CVD risk factor, many of which suffer from methodological or design weaknesses. The results show improvements in different health parameters, most of which were related to cardiovascular function: blood pressure, blood cholesterol levels, and atherosclerosis [18]. These results are interesting because arterial hypertension is frequently observed in combination with hypercholesterolemia, which is related to accelerated atherosclerosis [19].

In previous research projects, ABG extract obtained by the same production process used in the present work showed antioxidant [20], anti-inflammatory, and vascular effects in in vitro tests carried out in animal tissues [21]. Additionally, the consumption of this ABG by rodents with diet-induced severe metabolic syndrome improved metabolic and vascular alterations and had broad effects related to gene and protein levels involved in inflammation, fat metabolism, and food intake regulation [22]. However, the beneficial effects of the regular consumption of this ABG on CVD risk factors in humans remain undefined.

Accordingly, we hypothesized that daily administration of a well-characterized ABG extract with a standardized SAC yield produces favorable changes in CVD risk factors in individuals with moderate hypercholesterolemia.

Thus, the main aim of the present study was to assess the effect of daily intake of an ABG extract characterized by a high concentration of SAC in combination with dietary recommendations regarding CVD risk factors in individuals with moderate hypercholesterolemia.

## 2. Materials and Methods

### 2.1. Study Population

Subjects from the general population were enrolled by means of advertising in newspapers, tableaux advertisements, and social networks at the Hospital Universitari Sant Joan (HUSJ)-Eurecat, Reus, Spain, between 24 July and 4 September 2019. Among 75 subjects assessed for eligibility, 67 individuals with moderate hypercholesterolemia, according to current guidelines [23], were recruited. The inclusion criteria were age ≥ 18 years, LDL cholesterol (LDLc) levels ≥ 115 mg/dL, and willingness to provide informed consent before the initial screening visit. The exclusion criteria were body mass index (BMI) values ≥ 35 kg/m^2^; LDLc levels ≤ 115 and ≥190 mg/dL or current lipid-lowering treatment (i.e., drugs and functional foods); fasting blood glucose ≥ 126 mg/dL, verified two times; anemia (hemoglobin ≤ 13 g/dL in men and ≤12 g/dL in women); the use of multivitamin supplements, dietary supplements or nutraceutics, or phytotherapeutic products that interfere with the treatment under study; active smoking; adoption of a hypocaloric diet and/or pharmacological treatment for weight loss; the use of antihypertensive drugs; a diagnosis of chronic gastrointestinal disease; pregnancy or an intention to become pregnant; breastfeeding; current or past participation in a clinical trial or nutritional intervention study in the last 30 days prior to study enrollment; an inability to follow the study’s guidelines.

Participants signed informed consent prior to their participation in the study, which was approved by the Clinical Research Ethical Committee of the Institut d’Investigació sanitària Pere Virgili (Ref. CEIM: 022/2019), Reus, Spain. The protocol and trial were conducted in accordance with the Helsinki Declaration and Good Clinical Practice Guidelines of the International Conference of Harmonization (GCP ICH) and were reported as CONSORT Herbal Extension. The trial is registered at ClinicalTrials.gov: NCT04010565.

### 2.2. Experimental Design

A randomized, crossover, sustained, double-blind, and controlled intervention study was performed. The study participants were randomly divided into two groups of sequences. The randomization plan was generated using a website (www.randomization.com; accessed on 11 February 2019 at 12:26:02 PM CET) with 1:1 allocation using a random block. Participants, researchers, and the statistician remained blinded to the type of product administered throughout the study.

The participants were randomized depending on whether they started the study by taking 1 tablet per day containing ABG extract or a placebo for 6 weeks. The ABG extract was manufactured by Pharmactive Biotech Products, SL (Madrid, Spain) according to a proprietary process from garlic harvested in the area of Las Pedroñeras (Castilla-La Mancha, Spain), and the tablets were manufactured by Instant Procès (La Roca del Vallès, Spain) in February 2019. Each ABG tablet (550 mg) contained 250 mg of ABG extract, which provided 1.25 mg SAC/tablet plus 300 mg of excipients as shown in Table 1. Other minor components were 0.1 mg alliin and 0.03 μg furfurals, measured as 5-HMF per tablet. In the placebo tablets (550 mg), the garlic extract was replaced by maltodextrin. Intervention tablets were similar in appearance and smell, with natural vanilla flavoring used to confer a similar smell, and the tablets were differentiated only by a code assigned—111 or 222—by an independent researcher not involved in the study to guarantee blinding. The detailed compositions of the intervention products are described in Table 1.

During the intervention period and after enrollment, the participants were instructed to take 1 tablet per day containing ABG extract or the placebo at any time of the day and also to maintain their physical activity. Dietary habit recommendations based on cardioprotective dietary recommendations and instructions to avoid lipid-lowering and antihypertensive food consumption were given to the participants through nutritionist advice.

The participants’ adherence to their dietary habits throughout the study was assessed by a 3-day food record at baseline and after 6 weeks during both treatments.

### 2.3. Outcomes

All clinical outcomes were measured at baseline and after 3 and 6 weeks of intervention with both treatments.

Systolic blood pressure (SBP) and diastolic blood pressure (DBP) were measured twice after 2–5 min of rest with the participant in fasting conditions and in a seated position, with a one-minute interval between measurements. The mean values were used for statistical analyses. An automatic sphygmomanometer (OMRON HEM-907; Peroxfarma, Barcelona, Spain) was used. PP, which represents the force that the heart generates each time that it contracts, was determined by the difference between SBP and DBP [24].

Lipid profiles (total cholesterol (LDLc); HDL cholesterol (HDLc); triglycerides (TGs); non-esterified fatty acids (NEFAs)) and glucose were measured in serum by standardized enzymatic automated methods in an AU5800 clinical chemistry analyzer. Apolipoproteins (Apo) A-1 and B-100 as well as insulin were measured in serum by standardized immunoassay automated methods in a UniCel DxI 800 Access (Beckman Coulter, Inc., Nyon, Swiss). LDLc was calculated using the Friedewald formula [25].

Moreover, insulin sensitivity was calculated by the following formulas: according to HOMA-IR and QUICKI [26]: HOMA-IR = (fasting insulin µm/mL) + (fasting glucose mg/dL)/405. QUICKI = 1/(log(fasting insulin µU/mL) + log(fasting glucose mg/dL)).

Systemic inflammatory, oxidative, and vasodilatory biomarkers were also determined. Serum high-sensitivity C-reactive protein (hs-CRPs) and interleukin 6 (IL-6) were determined by automated high-sensitivity immunoassay methods in a UniCel DxI 800 Access device (Beckman Coulter, Inc.). Monocyte chemotactic protein-1 (MCP-1) and nitric oxide (NO) were measured in serum by ELISA kits (R&D Systems, Minneapolis, USA and Cayman Chemical, MI, USA, respectively). Plasma oxidized LDL (LDLox) was measured by an ELISA kit (Mercodia AB, Uppsala, Sweden).

Anthropometric and adiposity measures were also determined. Anthropometric measurements were conducted with the participants wearing lightweight clothing and no shoes. Waist circumference (WC) was measured at the umbilicus using a 150 cm anthropometric steel measuring tape. Body weight and body composition were obtained by a calibrated scale (Tanita SC 330-S; Tanita Corp., Barcelona, Spain). Height was measured using a wall-mounted stadiometer (Tanita Leicester Portable; Tanita Corp., Barcelona, Spain). BMI was calculated as the ratio between the measured weight (kg)/and the square of height (m). The conicity index was calculated as (WC (m)/(0.109 × square root of weight (kg)/height (m))) [27].

Dietary adherence: at baseline and at the end of each intervention, dietary compliance was monitored using 3 day dietary records and was confirmed in interviews with the nutritionist. In addition, adherence to the dietary recommendations was verified through a 24 h record by trained nutritionists at each follow-up visit.

### 2.4. Sample Size

The sample size was calculated using GPower 3.1 software considering the circulating levels of LDLc [28]. Thus, to detect a significant difference of 7.3 mg/dL in LDLc levels between the two interventions (i.e., ABG extract and placebo), assuming the values were consistent with the results reported in the available literature [29,30], the total number of individuals required considering a baseline LDLc level of 150 mg/dL with a standard deviation (SD) of 13.05, a statistical power of 80% and a confidence level of 95% by Student’s *t*-test was 52 individuals. If 15% of the participants were assumed to not complete the study, the number of individuals required to carry out the study was 60 (52 + 8).

Moreover, this study assessed the beneficial effects of AGB extract on several CVD biomarkers. Accepting an alpha risk of 0.05 and a beta risk of 0.2 in a two-sided test, 68 subjects were necessary to recognize statistically significant differences greater than or equal to 3.6 DBP units. The standard deviation was assumed to be 10 mmHg. A dropout rate of 10% was anticipated.

### 2.5. Statistical Analysis

Descriptive variables are expressed as the mean ± SD or the mean (95% confidence interval, CI). The data were analyzed by intention-to-treat (ITT).

The parametricity of the variables was examined, and logarithmic transformation of variables was performed if required. Differences in baseline characteristics among sequences of administration were assessed by ANOVA. Given that the study was a crossover study, data conversion from sequence to treatment was required. Intratreatment differences were assessed by a general linear model when three time points were involved (*p*-values are expressed in the tables) and by the ANCOVA model adjusted by age, sex, and the sequence of treatment administration when only two time points were involved (*p*-values included in the text).

Differences between treatments were assessed by ANCOVA adjusted by age, sex, and the sequence of treatment administration. For the variables for which an interaction with sex was observed in the ANCOVAs, the models were examined separately by sex.

Missing data were imputed by linear regression analyses with the exception of hs-CRP, where imputation was applied using the mean values due to the highly skewed distribution values.

## 3. Results

### 3.1. Study Population

**Figure 1:**
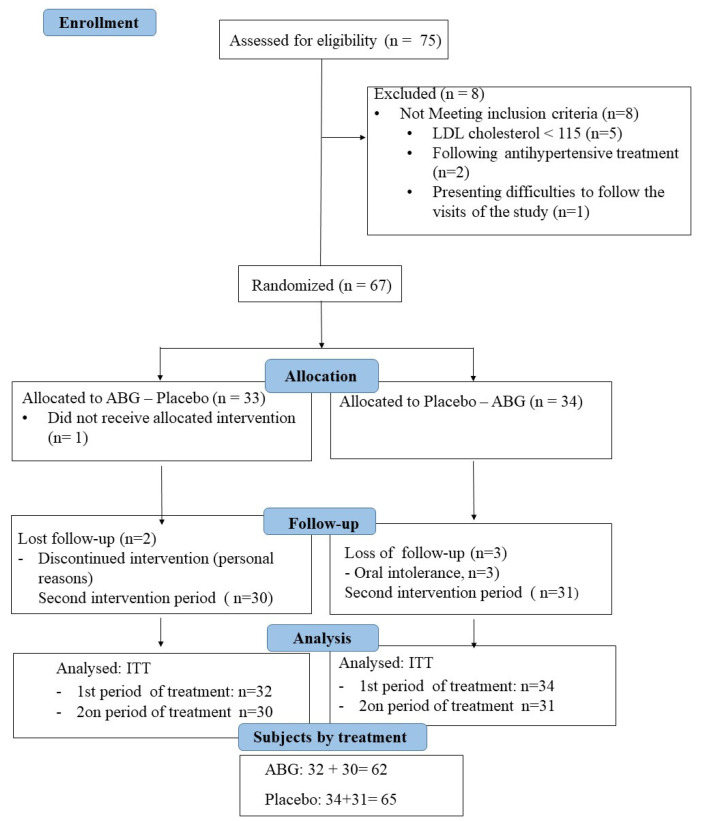
Participant flowchart. LDL, low density lipoprotein; ABG aged black garlic; ITT, intention-to-treat.

From the 75 eligible participants, 67 were randomized, and, finally, data from 62 participants in the ABG extract group and 65 participants in the placebo group were available. A CONSORT flowchart of the study is depicted in Figure 1.

According to the crossover design, 33 participants (mean age 53.7 ± 8.12 y; 18 females) were randomized in sequence 1 (ABG-Placebo), and 34 participants (52.7 ± 8.59 y; 17 females) were randomized in sequence 2 (Placebo-ABG). Table 2 describes the baseline characteristics of the participants by the sequence of product administration; no differences were observed.

### 3.2. Compliance, Adverse Events, and Product Tolerance

The bottles with the tablets were returned after each study phase. Tablet consumption compliance was (mean ± SD) 96.51 ± 8.82%, reflecting a high level of adherence considering a consumption of >80% to be an acceptable level of adherence.

No statistically significant differences were found between the two interventions with respect to the adverse events reported. The ABG product was well tolerated.

### 3.3. Cardiovascular Disease Risk Factors

#### 3.3.1. Blood and Pulse Pressure

Table 3 shows the changes in BP, PP, and cardiac frequency after 6 weeks of each treatment. No significant differences were observed after 3 weeks of intervention with either of the treatments.

At 6 weeks, the decrease in DBP after consumption of the ABG extract reached significance compared to changes observed after placebo consumption (*p* = 0.007). After ABG intake, DBP also decreased, reaching borderline significance (*p* = 0.083) compared to baseline.

The observed decrease in DBP had a different pattern between sexes and/or by categories of DBP at baseline. Stratification by sex and by categories of DBP according to the median values at the initial visit showed that the decrease in DBP after ABG consumption compared with placebo consumption was particularly significant in men and in individuals with a DBP > 75 mm Hg (*p* < 0.05) (Table 4).

No intra- or intertreatment differences were observed in SBP, PP, or cardiac frequency.

#### 3.3.2. Lipid Profile, Apos, and NEFAs

No significant differences were observed after 3 weeks of intervention with either of the treatments. The changes in the cardiovascular risk lipid profile at 6 weeks after treatment and stratified by categories of the median values are shown in Table 5.

At week 6, compared to those at baseline, total cholesterol levels showed a quadratic decreasing trend after ABG treatment (*p* = 0.047), which remained when the total cholesterol concentrations were categorized at total cholesterol values ≤ 242 mg/dL. No intertreatment differences were observed in this parameter.

Table 6 shows the changes in Apos and NEFAs. At 6 weeks of intervention, no intra- or intertreatment differences were observed.

#### 3.3.3. Homeostatic Glucose Profile Biomarkers and Systematic Inflammatory, Oxidative, and Vasodilatory Biomarkers

The changes in the glucose profile and inflammatory, oxidative, and vasodilatory biomarkers at 6 weeks are shown in Supplementary Materials Tables S1 and S2. At 6 weeks of intervention, no intra- or intertreatment differences were observed. Moreover, no significant differences were observed after 3 weeks of intervention with either of the treatments.

#### 3.3.4. Anthropometric Measures

Table 7 shows the changes in anthropometric and adiposity measures at 6 weeks of intervention in the whole population; no intra- or intertreatment differences were observed.

The changes in anthropometric and adiposity measures at 6 weeks of intervention by sex are shown in Table 8. No intra- or intertreatment differences were observed in either males or women in the variables in the ANCOVA model. The decrease in the conicity index in women after ABG treatment reached borderline significance (*p* = 0.093) vs. changes in men.

No significant differences were observed after 3 weeks of intervention with either of the treatments.

#### 3.3.5. Dietary Adherence

Table 9 shows the changes in dietary composition (i.e., energy, nutrients, fiber, and alcohol) after 6 weeks of intervention. At the end of the two intervention periods, compared to that at baseline, a significant reduction in monounsaturated fatty acid (MUFA) consumption, both when expressed as a % of energy or grams, was observed after both treatments (*p* < 0.05), without intertreatment differences. Furthermore, a significant decrease in polyunsaturated fatty acids (PUFAs) (*p* = 0.029), expressed as a % of energy, and a significant increase in total fat (% of energy) (*p* = 0.030) were observed after placebo treatment as well as a borderline decrease in grams of protein (*p* = 0.08) after this treatment without intertreatment differences.

## 4. Discussion

In the present study, which was carried out with moderately hypercholesterolemic subjects, daily consumption of aged garlic extract containing 1.25 mg SAC (ABG extract) improved DBP compared with placebo consumption, particularly in men and in individuals with a baseline DBP higher than 75 mm Hg, by a mean of (95% CI) −4.82 (−9.7; −0.02) mm Hg.

Hypertension affects 30% of adults worldwide [31] and is the leading preventable risk factor for CVD and all-cause mortality. In the present study, the observed reduction in DBP by ABG extract was similar to the effects of dietary approaches, including the effects of the Dietary Approaches to Stop Hypertension (DASH) diet on BP, which significantly reduced DBP by 3.54 mm Hg (95% CI: −4.29; −2.79) [32]. Other diets, such as the Mediterranean, low-carbohydrate, paleolithic, high-protein, low-glycemic index, low-sodium, and low-fat dietary approaches, have produced significant reductions in DBP from −4.85 to −1.27 mm Hg [33,34].

Although SBP elevation has a greater effect on outcomes, both systolic and diastolic hypertension independently influences the risk of adverse cardiovascular events regardless of the definition of hypertension (≥140/90 or ≥130/80 mm Hg) [35].

The importance of the risk of death from ischemic heart disease and stroke doubles with every 10 mmHg increases in DBP among people aged 40–89 years. Thus, reducing DBP by 5 mmHg results in a 40% lower risk of death from stroke and a 30% lower risk of death from ischemic heart disease or other vascular death [35].

To the best of our knowledge, this is the first report of the DBP-reducing effect of an ABG extract in a population where the intervention strategies were based on diet and maintaining a healthy lifestyle without the mandatory use of drugs [23], and the effects were observed following an easy treatment schedule of one optimized ABG extract tablet consumed daily.

Preliminary clinical trials show the antihypertensive effect of garlic extracts other than ABG extract [18,36]. Nevertheless, previous evidence differs from our findings by two factors [36,37,38,39,40,41]. First, all previous studies in humans involved the use of proprietary aged garlic generated by soaking sliced raw garlic in 20% ethanol for up to 20 months at ambient temperature [37]. Those clinical trials were performed in heterogeneous groups of patients aged over 60 years who were obese or overweight, suffered from hypertension, even uncontrolled hypertension, and comorbidities, and who were often receiving diverse pharmacological treatments, and most of them had moderate to very high CVD risk. In all cases, the improved parameter was SBP, with scattered results regarding DBP [38,40,41,42,43].

In one report, the antihypertensive effect was reported after 12 week consumption of aged garlic containing 2.4 mg SAC, and the effect on SBP was significant only in those with a baseline SBP > 150 mm Hg [38]. In another study, with the intake of two capsules of the aged extract containing 1.2 mg SAC/day for 12 weeks, a subgroup of responders showed a more reduced SBP when the baseline SBP was over 150 mm Hg. The DBP response was significant only when the starting mean DBP value was 93 mm Hg [42]. The BP reduction was replicated in another 12 week clinical trial performed with fewer participants and a baseline difference between participants allocated to a placebo and aged garlic groups after randomization [43]. In this case, at 12 weeks, a significant reduction in SBP was reported in the aged garlic-consuming group (mean baseline SBP of 153 mm Hg) compared to the placebo-consuming group [43]. In particular, the highest SBP statistical reduction was identified in a subgroup of participants taking standard BP medication compared to participants not on BP treatment (*n* = 7/9) of 4.9 ± 5.4 mm Hg at baseline [43]. Moreover, the authors observed a significant reduction in DBP (mean baseline DBP of 93 mmHg) compared to that in the placebo group. The highest DBP reduction was detected in participants without antihypertensive treatment in comparison with participants taking a placebo [43].

In a dose–response study performed with normo- or hypercholesterolemic hypertensive patients, most of whom were treated with one or more antihypertensive drugs, with a mean age close to 70 years and a mean BMI close to 30 kg/m^2^, the participants consumed aged garlic at 0.6, 1.2, or 2.4 mg SAC/day (between one and four capsules daily) for 12 weeks [40]. In the end, SBP significant differences were found only in those consuming 1.2 mg SAC/day compared to the placebo [40], whereas no differences were found in DBP [40]. In contrast, the referred aged garlic extract of 1.2 g/day for 12 weeks did not affect blood pressure in participants suffering from metabolic syndrome (obesity, low HDLc, and glucose resistance) [41].

The differences in how aged garlic extracts and ABG consumed in the present work modulate blood pressure can be explained by several reasons: (1) In previous studies, aged garlic extracts were assayed in humans with different ages, BMIs, and/or marked differences in basal medical concerns and health status. Nevertheless, in the present study, the participants presented moderate hypercholesterolemia and optimal or elevated blood pressure levels. (2) Dietary intake monitoring was not performed in clinical trials investigating different aged garlic extracts, whereas in the present study, 3 day dietary records were employed. (3) The chemical compositions and the amounts of active ingredients differed between the former aged garlic extracts and the ABG. Although the ABG consumed in the present study and the aged garlic used in other studies [40,41,43] have shown similar effects at the same SAC doses, both extracts differed in the manufacturing process due to the presence of substances (i.e., the ABG extract used in this study did not contain furfurals), and other differences may be due to the chemical background of the garlics and the pre-harvesting conditions [44]. Additionally, the surplus of organosulfurs other than SAC was dissimilar in both extracts.

The suggested mechanisms of action by which aged garlic could modulate blood pressure involve antioxidant activity by organosulfur compounds, such as SAC, in addition to allicin-derived polysulfides and the NOx and hydrogen sulfide signaling pathways, the regulation of transcription factors involved in hypertension, and ACE regulation [39,45]. Some of them have already been identified in the current ABG extract: antioxidant properties in vitro, improvement of vasodilation and levels of iNOS and eNOS, and regulation of the expression and levels of proinflammatory TNF-α [20,21,22].

In the present clinical study, no other significant clinical results were observed in relation to lipid profiles, glucose profiles, anthropometric measures and inflammation, and oxidative and vasodilatory biomarkers. However, in previous in vitro studies with ABG extract similar to that used in the present study, the presence of 75 μg/mL improved heart and vascular functions in rat tissues [21]. In rats suffering from metabolic syndrome induced by a high fat-high fructose diet, the administration of 0.28 mg SAC/kg body by gavage weight daily curved the glucose, fat, and cholesterol metabolic disturbances produced by the diet and attenuated obesity-induced vasoconstriction [22].

Therefore, previously observed effects in animals appear to not be reproducible in humans; however, more studies are needed to assess the effects of ABG extract on other CVD risk factors in humans including patients who suffer inflammatory pathologies.

Considering the beneficial effects of the ABG extract on DBP, future clinical studies focusing on the antihypertensive effects in subjects with elevated blood pressure or grade 1 hypertension (SBP = 130–159 mm Hg and/or DBP = 85–99 mm Hg) are highly encouraged. Thus, ABG extract may promote healthy lifestyle choices to prevent or delay increased blood pressure even though lifestyle modifications are often implemented after antihypertensive treatment initiation [46]. Moreover, as blood pressure is under circadian influence, in future studies, volunteers could be instructed to always take the optimized aged garlic extract at the same time of the day, to assess if the effects on BP were improved. However, the ideal time of the day to take hypotensive medication in order to achieve the best control of BP remains in discussion [47].

In addition, the analysis of some biomarkers of consumption or exposure to ABG extract would also be recommended to provide a more precise measure of ABG compound intake. This approach will allow us to cover the interface between the intake of ABG extract and the ultimate effects of the bioactive components of the extract (SAC) on the physiological and health status of the consumers.

The changes detected in DBP after ABG extract intake cannot be related to dietary modifications, as no significant differences were observed between the ABG intervention and the placebo at 6 weeks. In addition, as the anthropometric parameters (i.e., BMI and WC) did not change during the study, the results observed for DBP can be specifically attributed to ABG consumption.

One of the strengths of the present study is its crossover design, minimizing the interference of interindividual response variation, as each subject acts as the corresponding control. Moreover, the design as a randomized, placebo-controlled clinical trial is able to provide the first level of scientific evidence using a product without ABG as a placebo. The main limitation of the present study was that the biomarker for ABG consumption remains undetermined.

## 5. Conclusions

In conclusion, at 6 weeks, the consumption of 250 mg of ABG extract with 1.25 mg of SAC significantly decreased DBP in moderately hypercholesterolemic subjects. The potential beneficial effects of ABG could be better observed in men and in nonoptimal DBP populations. Further studies performed in subjects with elevated blood pressure or grade 1 hypertension are needed to elucidate the specific contribution of SAC to the promising antihypertensive effects observed with this aged black garlic extract.

## Figures and Tables

**Table 1:** Product compositions.

Description	Aged Black Garlic Extract Weight (mg Per Tablet)	Placebo Weight (mg Per Tablet)
Pharmactive’s aged black garlic Extract	250 (1.25 mg SAC)	-
Maltodextrin	-	250
Microcrystalline cellulose	90	90
Dicalcic phosphate	157	157
Sodium croscarmellose	10	10
Magnesium stearate	7	7
Sodium alginate	3.06	3.06
Stearic acid	0.03	0.03
Oleic acid	1.54	1.54
Medium-chain triglycerides (MCTs)	2.80	2.80
Ethylcellulose	13.17	13.17
Hydroxypropyl methylcellulose (HPMC)	4.86	4.86
Hydroxypropyl cellulose (HPC)	4.86	4.86
Talc	2.88	2.88
Titanium dioxide	1.80	1.80
Natural Vanilla flavor	1.00	1
Total	550	550

At the end of week 6 of the study, when the first treatment was completed, a washout period of 3 weeks was carried out, and then the treatment was exchanged, which was conducted for another 6 weeks; the total duration of the study for each participant was 15 weeks (6 weeks + 3 weeks + 6 weeks).

**Table 2:** Baseline characteristics of participants by sequence of administration.

	Sequence 1 (*n* = 33)	Sequence 2 (*n* = 34)	*p*-Value
Age, y	53.7 ± 8.12	52.7 ± 8.59	0.609
Female, %	54.5	50.0	0.449
Systolic blood pressure, mm Hg	124 ± 17.7	125 ± 15.0	0.784
Diastolic blood pressure, mm Hg	76 ± 11.4	76 ± 9.77	0.854
Pulse pressure, mm Hg	48 ± 8.47	50 ± 10.1	0.489
Weight, kg	72 ± 14.0	72 ± 11.4	0.991
Body mass index, kg/m^2^	25.7 ± 3.06	25.8 ± 3.23	0.993
Waist circumference, cm	91 ± 8.74	90 ± 9.55	0.644
Waist/height	0.55 ± 0.05	0.54 ± 0.06	0.561
Conicity index	1.28 ± 0.08	1.26 ± 0.08	0.341
Fat mass, %	28.1 ± 8.56	27.3 ± 8.98	0.727
Fat mass, kg	20.9 ± 6.62	20.2 ± 6.66	0.675
Lean mass, kg	51.1 ± 11.0	51.4 ± 9.83	0.901
Muscle mass, kg	48.5 ± 10.4	48.8 ± 9.37	0.900
Bone mass, kg	2.58 ± 0.53	2.60 ± 0.47	0.881
Total water, %	49.1 ± 4.46	50.2 ± 5.83	0.411
Total water, kg	35.4 ± 7.65	36.0 ± 7.33	0.740

Sequence 1: AGB followed by placebo; Sequence 2: placebo followed by AGB. AGB, aged black garlic. Data are expressed as the mean ± standard deviation.

**Table 3:** Blood and pulse pressure and cardiac frequency at baseline and after 6 weeks of intervention.

	Intervention				Change Intertreatments	
Variable	ABG		Placebo		ABG vs. Placebo	
	Baseline	Change at 6 Weeks	Baseline	Change at 6 Weeks	Mean	*p*-Value
					(95% CI)	
SBP, mm Hg	124 ± 17.9	1.32 (−4.1; 6.7)	121 ± 18.4	2.84 (−2.4; 8.1)	−1.51 (−9.1; 6.1)	0.694
DBP, mm Hg	75 ± 10.3	−1.85 * (−3.7; 0.001)	74 ± 9.80	1.77 (−0.10; 3.6)	−3.623 (−6.2; −0.99)	0.007
PP, mm Hg	48 ± 14.9	1.74 (−3.1; 6.6)	47 ± 14.7	−0.363 (−5.1; 4.4)	−2.00 (−8.9; 4.7)	0.544
CF, beats/min	66 ± 17.8	−1.32 (−2.4; 5.0)	66 ± 13.7	−0.422 (−6.9; 3.5)	−1.75 (−8.9; 4.7)	0.507

ABG; aged black garlic; change, change from baseline; SBP, systolic blood pressure; DBP; diastolic blood pressure; PP, pulse pressure (SBP-DBP); CF, cardiac frequency. Data are expressed as the mean ± standard deviation or mean (95% Confidence Interval, CI). ANCOVA model adjusted by sex, age, sequence of treatments administration, and baseline values. * *p* = 0.083, non-adjusted *p*-value by Student’s *t*-test for related samples.

**Table 4:** Changes in diastolic blood pressure at 6 weeks of intervention by categories of diastolic blood pressure at V0 (cut off for the median value) and by gender.

Intervention					Change Intertreatments	
Variable	ABG		Placebo		ABG vs. Placebo	
	Baseline	Change	Baseline	Change	Mean (95% CI)	*p*-Value
Gender						
Male	80 ± 9.4	−3.17 (−6.4; 0.03)	76 ± 9.9	2.71 (−0.59; 6.0)	−5.85 (−10.5; −1.3)	0.013
Women	71 ± 9.9	−0.579 (−2.5; 1.4)	72 ± 9.4	0.936 (−0.98; 2.8)	−0.151 (−4.2; 1.2)	0.271
Categories in DBP at V0						
DBP ≤ 75 mm Hg	69 ± 6.7	0.061 (−2.1; 2.2)	69 ± 6.3	2.86 (0.70; 5.0)	−2.80 (−5.8; 0.23)	0.070
DBP > 75 mm Hg	83 ± 8.8	−4.42 (−6.2; −0.99)	80 ± 9.6	0.400 (−3.0; 3.8)	−4.82 (−9.7; −0.02)	0.049

ABG, aged back garlic; change, change from baseline; DBP, diastolic blood pressure. Data are expressed as the mean ± standard deviation or the mean (95% Confidence Interval, CI). V0, first visitation. ANCOVA model adjusted by sex, age, sequence of treatments administration, and baseline values.

**Table 5:** Changes in the lipid profile at 6 weeks after treatments by categories of variables according to the median value at baseline.

Intervention								
Variable		ABG			Placebo		Change Intertreatments	
	Baseline	Change	P for Trend	Baseline	Change	P for Trend	Mean (95% CI)	*p*-Value
Total cholesterol, mg/dL								
Global	243 ± 32	0.865 (−4.1; −5.9)	0.047 ^†^	240 ± 30	2.15 (−3.1; 7.4)	0.421 *	−1.29 (−8.6; 6.0)	0.727
≤242 mg/dL	219 ± 19	5.52 (−1.3; 12)	0.028 ^†^	218 ± 22	6.90 (−1.9; 18)	0.120 *	0.914 (−8.7; 10)	0.849
>242 mg/dL	267 ± 24	−3.94 (−11; 3.4)	0.283 *	263 ± 21	−0.329 (−8.6; 8.0)	0.872 ^†^	−3.49 (−7.7; 15)	0.535
LDLd, mg/dL								
Global	165 ± 24	−0.390 (−4.1; 3.3)	0.011 ^†^	165 ± 22	0.560 (−4.8; 3.7)	0.279 ^†^	0.156 (−5.8; 5.5)	0.956
≤166 mg/dL	151 ± 14	3.33 (−1.1; 7.6)	0.143 *	147 ± 19	0.718 (−4.7; 6.1)	0.131 ^†^	2.69 (−9.6; 4.2)	0.438
>166 mg/dL	188 ± 17	−8.20 ^‡^ (−15; −1.6)	0.017 * 0.014 ^†^	182 ± 18	−2.55 (−9.8; 4.7)	0.475 ^†^	−6.28 (−16; 3.1)	0.184
LDL (Friedewald), mg/dL								
Global	162 ± 26	1.47 (−2.7; 5.6)	0.049 ^†^	161 ± 25	1.91 (−3.3; 7.2)	0.308 ^†^	−0.258 (−6.6; 7.1)	0.941
≤162 mg/dL	148± 17	4.50 (−0.41; 9.41)	0.071 *	148 ± 19	4.11 (−2.6; 11)	0.221 *	0.729 (−7.9; 9.3)	0.866
>162 mg/dL	186 ± 18	−3.79 (−11; 3.7)	0.063 ^†^	184 ± 17	−1.99 (−11; 6.9)	0.607 ^†^	−1.85 (−13; 9.5)	0.744
HDL, mg/dL								
Global	56.9 ± 11.4	0.005 (−0.99; 1.0)	0.354 ^†^	55.0 ± 10.9	1.02 (−0.41; 2.5)	0.159 *	−0.695 (−2.5; 1.1)	0.492
≤55.5 mg/dL	49.3 ± 7.86	−0.045 (−1.4; 1.3)	0.136 ^†^	48.2 ± 7.23	1.01 (−0.90; 2.9)	0.124 ^†^	−1.49 (−3.7; 0.70)	0.178
>55.5 mg/dL	65.3 ± 8.32	0.061 (−1.4; 1.6)	0.816 ^†^	62.8 ± 9.19	1.04 (−1.2; 3.3)	0.360 *	0.105 (−2.9; 3.1)	0.945
Total Cholesterol/ HDL								
Global	4.41 ± 0.92	0.006 (−0.09; 0.10)	0.008 ^†^	4.48 ± 0.82	−0.023 (−0.16; 0.12)	0.239 ^†^	0.033 (−0.13; 0.20)	0.694
≤4.44	3.73 ± 0.45	0.062 (−0.03; 0.16)	0.200 *	3.86 ± 0.51	−0.022 (−0.22; 0.18)	0.358 ^†^	0.074 (−0.15; 0.29)	0.506
>4.44	5.10 ± 0.74	−0.052 (−0.22; 0.12)	0.004 ^†^	5.06 ± 0.60	−0.024 (−0.23; 0.18)	0.443 ^†^	−0.023 (−0.30; 0.25)	0.864
LDLd/HDL								
Global	3.02 ± 0.72	−0.013 (−0.08; 0.05)	0.001 ^†^	3.09 ± 0.64	−0.049 (−0.15; 0.05)	0.168 ^†^	0.156 (−5.8; 5.5)	0.956
≤3.07	2.47 ± 0.33	0.065 (−0.01; 0.14)	0.105 *	2.60 ± 0.41	−0.120 (−0.25; 0.07)	0.062 *	0.077 (−0.09; 0.24)	0.362
>3.07	3.58 ± 0.58	−0.114 (−0.24; 0.01)	0.071 * 0.002 ^†^	3.55 ± 0.46	0.019 (−0.14; 0.18)	0.356 ^†^	−0.05 (−0.21; 0.16)	0.782
LDL(Fried)/HDL								
Global	2.95 ± 0.72	0.038 (−0.04; 0.12)	0.002 ^†^	3.00 ± 0.63	0.001 (−0.11; 0.11)	0.089 ^†^	0.041 (−0.09; 0.18)	0.551
≤2.94	2.43 ± 0.40	0.072 (−0.01; 0.15)	0.084 ^†^	2.52 ± 0.41	−0.036 (−0.19; 0.12)	0.154 ^†^	0.089 (−0.09; 0.27)	0.319
>2.94	3.49 ± 0.56	−0.021 (−0.15; 0.11)	0.002 ^†^	3.45 ± 0.45	0.035 (−0.14; 0.21)	0.280 ^†^	−0.021 (−0.22; 0.18)	0.840
TG (log), mg/dL								
Global	2.04 ± 0.18	−0.031 ^‡^ (−0.06; − 0.001)	0.042 *	2.05 ± 0.19	−0.016 (−0.05; 0.02)	0.419 *	−0.016 (−0.03; 0.06)	0.531
≤2.00	1.90 ± 0.09	−0.011 (−0.05; 0.03)	0.568 *	1.91 ± 0.09	0.024 (−0.04; 0.09)	0.438 *	−0.021 (−0.10; 0.06)	0.603
>2.00	2.16 ± 0.15	−0.049 (−0.09; −0.002)	0.040 *	2.15 ± 0.13	−0.037 (−0.08; 0.07)	0.095 *	−0.019 (−0.05; 0.09)	0.578
Log (TG/HDL)								
Global	0.30 ± 0.23	−0.033 ^‡^ (−0.06; −0.001)	0.046 *	0.31 ± 0.23	−0.023 (−0.07; 0.02)	0.309 *	−0.020 (−0.07; 0.04)	0.640
≤0.24	0.12 ± 0.11	−0.010 (−0.05; 0.03)	0.613 *	0.14 ± 0.12	0.009 (−0.06; 0.08)	0.789 *	−0.020 (−0.10; 0.06)	0.610
≤0.24	0.47 ± 0.17	−0.055 ^‡^ (−0.11; −0.003)	0.038 *	0.49 ± 0.17	−0.055 (−0.12; 0.006)	0.073 *	−0.004 (−0.08; 0.07)	0.909

ABG, aged black garlic; change, change from baseline; w, weeks; LDL, low-density lipoproteins; d, directly measured; fried, calculated by Friedewald formula; HDL, high-density lipoproteins; TGs, triglycerides. Data are expressed as the mean ± standard deviation or the mean (95% Confidence Interval, CI). Intra-treatment comparison by general linear model. P for: * quadratic trend; ^†^ linear trend; ^‡^ *p* < 0.05 vs. the baseline. Intertreatment comparisons by ANCOVA model adjusted by age, sex, and order.

**Table 6:** Changes in apolipoproteins, their ratios, and NEFA after 6 weeks of intervention.

	Intervention				Change Intertreatments	
Variable	ABG		Placebo		ABG vs. Placebo	
	Baseline	Change	Baseline	Change	Mean (95% CI)	*p*-Value
ApoA1, mg/dL	144 ± 20.2	−2.25 (−4.6; 0.14)	141 ± 0.3	0.038 (−2.3; 2.4)	−2.29 (−5.6; 1.1)	0.178
ApoB100, mg/dL	104 ± 15.4	−0.117 (−3.0; 2.7)	104 ± 14.2	−0.516 (−3.4; 2.3)	0.338 (−4.4; 3.4)	0.868
ApoB100/ApoA1	0.74 ± 0.16	0.012 (−0.01; 0.03)	0.75 ± 0.14	0.000 (−0.02; 0.02)	0.012 (−0.02; 0.04)	0.459
TG/ApoB100	1.13 ± 0.50	−0.082 (−0.20; 0.03)	1.16 ± 0.52	−0.058 (−0.17; 0.05)	−0.024 (−0.18; 0.13)	0.766
NEFA, nmol/L	0.39 ± 0.14	−0.005 (−0.05; 0.04)	0.40 ± 0.14	−0.020 (−0.06; 0.02)	0.015 (−0.05; 0.08)	0.637

ABG, aged black garlic; change, change from baseline; TGs, triglycerides; NEFA, non-esterified fatty acids. Data are expressed as the mean ± standard deviation or the mean (95% Confidence Interval, CI). ANCOVA model adjusted by sex, age, and sequence of treatment administration.

**Table 7:** Changes in anthropometric and adiposity measures at 6 weeks of intervention.

Intervention					Change Intertreatments	
Variable	ABG		Placebo		ABG vs. Placebo	
	Baseline	Change	Baseline	Change	Mean (95% CI)	*p*-Value
Weight, kg	71.5 ± 12.5	0.130 (−0.44; 0.70)	71.4 ± 12.4	−0.219 (−0.78; 0.34)	0.348 (−1.1; 0.46)	0.393
BMI, kg/m^2^	25.7 ± 3.13	1.01 (−0.27; 2.3)	25.9 ± 3.29	−0.138 (−1.4; 1.1)	1.14 (−2.9; 0.66)	0.213
Waist circumference, cm	91 ± 8.93	−0.149 (−1.2; 0.89)	90 ± 9.72	0.283 (−0.76; 1.03)	0.432 (−1.0; 1.9)	0.563
Waist/Height	0.55 ± 0.05	−0.002 (−0.009; 0.005)	0.54 ± 0.05	0.002 (−0.005; 0.009)	0.004 (−0.006; 0.01)	0.403
Conicity index	1.27 ± 0.06	−0.003 (−0.02; 0.01)	1.26 ± 0.08	0.006 (−0.01; 0.02)	−0.009 (−0.01; 0.03)	0.410
Fat mass, %	28.7 ± 7.32	0.200 (−1.12; 1.52)	28.9 ± 8.09	−0.596 (−1.92; 0.73)	0.796 (−1.01; 2.67)	0.402
Fat mass, kg	20.3 ± 6.67	1.06 (−0.42; 2.5)	21.2 ± 7.68	−0.474 (−1.98; 1.0)	1.53 (−3.6; 0.58)	0.153
Lean mass, kg	51.4 ± 10.9	−1.052 (−2.7; 0.64)	51.2 ± 10.3	−1.020 (−2.7; 0.66)	−0.032 (−2.4; 2.3)	0.979
Muscle mass, kg	48.8 ±10.4	−0.362 (−1.5; 0.80)	48.3 ± 10.3	−0.058 (−1.2; 1.1)	−0.304 (−1.9; 1.3)	0.713
Bone mass, kg	2.59 ± 0.52	−0.013 (−0.07; 0.05)	2.58 ± 0.50	−0.015 (−0.07; 0.04)	0.002 (−0.08; 0.08)	0.964
Total water, %	49.6 ± 5.38	−0.142 (−2.1; 1.8)	49.3 ± 5.50	0.979 (−0.90; 2.9)	−1.121 (−1.6; 3.8)	0.412
Total water, kg	35.6 ± 7.74	0.325 (−0.66; 1.3)	35.3 ± 7.39	0.221 (−0.75; 1.2)	−0.105 (−1.5; 1.3)	0.881

ABG, aged bblack garlic; BMI, body mass index (weight in kg/(height in meters))^2^; waist/height, waist (cm)/height (cm) ratio; change, change from baseline. Data are expressed as the mean ± standard error or the mean (95% Confidence Interval, CI). ANCOVA model adjusted by sex, age, basal values, and sequence of treatment administration.

**Table 8:** Changes in anthropometric and adiposity measures at 6 weeks of intervention by gender.

Intervention					Change Intertreatments	
Variable	ABG		Placebo		ABG vs. Placebo	
	Baseline	Change	Baseline	Change	Mean (95% CI)	*p*-Value
Waist circumference, cm						
Total	100 ± 8.93	−0.149 (−1.2; 0.89)	90 ± 9.72	0.283 (−0.76; 1.03)	0.432 (−1.0; 1.9)	0.563
Men (*n* = 32)		0.513 (−1.4; 2.4)		1.26 (−0.62; 3.1)	0.753 (−1.9; 3.4)	0.574
Women (*n* = 35)		−0.755 (−1.8; 0.28)		−0.615 (−1.6; 0.42)	0.139 (−1.3; 1.6)	0.849
Waist/Height						
Total	0.55 ± 0.05	−0.002 (−0.009; 0.005)	0.54 ± 0.05	0.002 (−0.005; 0.009)	0.004 (−0.006; 0.01)	0.403
Men	0.55 ± 0.05	0.003 (−0.008; 0.01)	0.54 ± 0.05	0.008 (−0.003; 0.02)	0.005 (−0.01; 0.02)	0.557
Women	0.54 ± 0.05	−0.007 (−0.01; 0.000)	0.54 ± 0.06	−0.004 (−0.01; 0.003)	0.003 (−0.007; 0.01)	0.533
Conicity index						
Total	1.27 ± 0.06	−0.003 (−0.02; 0.01)	1.26 ± 0.08	0.006 (−0.01; 0.02)	−0.009 (−0.01; 0.03)	0.410
Men	1.28 ± 0.06	0.008 (−0.02; 0.04)	1.26 ± 0.10	0.016 (−0.01; 0.04)	−0.008 (−0.03; 0.05)	0.678
Women	1.27 ± 0.06	−0.014 * (−0.03; 0.004)	1.26 ± 0.07	−0.003 (−0.02; 0.01)	−0.011 (−0.01; 0.04)	0.405
Lean mass, kg	(*p* = 0.045)					
Total	51.4 ± 10.9	−1.052 (−2.74; 0.64)	51.2 ± 10.3	−1.020 (−2.70; 0.66)	−0.032 (−2.41; 2.35)	0.979
Men	60.7 ± 7.76	0.149 (−2.02; 2.31)	59.8 ± 7.15	−0.856 (−2.99; 1.27)	1.006 (−2.03; 4.04)	0.510
Women	43.2 ± 5.34	−2.124 (−4.67; 0.42)	43.2 ± 5.07	−1.161 (−3.70; 1.38)	−0.962 (−2.63; 4.56)	0.595

ABG, aged black garlic; waist/height, waist (cm)/height (cm) ratio; change, change from baseline. Data are expressed as the mean ± standard error or the mean (95% Confidence Interval, CI). ANCOVA model adjusted by sex, age, basal values, and sequence of treatment administration. * *p* = 0.093 vs. men.

**Table 9:** Energy, nutrients, fiber, and alcohol after 6 weeks of intervention.

	ABG	*p*-Value	Placebo	*p*-Value	P for Difference *
Energy, kcal/day Baseline 6 weeks	2031 ± 579	2004 ± 665 0.734	1950 ± 543 2015 ± 560	0.321	0.381
CHO, % energy Baseline 6 weeks	37.1 ± 8.7 36.7 ± 8.8	0.628	36.9 ± 6.8 36.0 ± 6.4	0.251	0.687
CHO, grams Baseline 6 weeks	186 ± 62 188 ± 83	0.822	179 ± 54 179 ± 53	0.995	0.854
Protein, % energy Baseline 6 weeks	16.7 ± 3.2 16.9 ± 4.7	0.759	17.0 ± 4.3 17.0 ± 2.9	0.930	0.864
Protein, grams Baseline 6 weeks	81.9 ± 23.9 82.3 ± 27.3	0.895	79.0 ± 24.2	84.2 ± 22.8 0.080	0.225
Total fat, % energy Baseline 6 weeks	41.8 ± 6.8 41.5 ± 7.0	0.769	41.8 ± 6.5 43.9 ± 7.3	0.030	0.102
Total fat, grams Baseline 6 weeks	96.9 ± 34.6 95.7 ± 39.3	0.818	96.3 ± 40.3 97.8 ± 34.3	0.446	0.776
SFA, % energy Baseline 6 weeks	11.9 ± 3.8 11.8 ± 3.4	0.793	12.0 ± 3.5 12.1 ± 3.2	0.831	0.716
SFA, grams Baseline 6 weeks	27.5 ± 11.8 27.7 ± 14.1	0.945	26.9 ± 13.0 28.1 ± 12.7	0.451	0.697
MUFA, % energy Baseline 6 weeks	23.4 ± 6.8 19.0 ± 5.0	<0.001	23.8 ± 5.7 20.2 ± 3.6	<0.001	0.463
MUFA, grams Baseline 6 weeks	55.1 ± 24.5 44.0 ± 19.2	0.003	52.1 ± 20.8 45.6 ± 15.2	0.010	0.324
PUFA, % energy Baseline 6 weeks	7.6 ± 2.1 7.1 ± 1.9	0.142	7.7 ± 2.3 7.0 ± 2.2	0.029	0.699
PUFA, grams Baseline 6 weeks	17.8 ± 8.0 16.0 ± 7.0	0.114	16.9 ± 7.3 16.4 ± 8.7	0.680	0.443
Fiber, g/day					
Baseline 6 weeks	20.2 ± 9.2 20.3 ± 9.6	0.958	20.4 ± 11.3 19.6 ± 8.0	0.259	0.606
Alcohol, g/day Baseline 6 weeks	5.8 (2.2–13.3) 5.3 (1.2–14.0)	0.866	8.2 (1.1–17.5) 7.9 (0.00–17.8)	0.507	0.947

ABG, aged black garlic; CHOs, carbohydrates; SFAs, saturated fatty acids; MUFAs, monounsaturated fatty acids; PUFAs, polyunsaturated fatty acids. Data are expressed as the mean ± standard deviation; median (25–75th percentile). Intra-treatment comparisons by Student’s *t*-test and Wilcoxon test for related samples. * *p*-Value for differences among treatments. ANOVA and Wilcoxon tests.

## Data Availability

The data presented in this study are available upon request from the corresponding author.

## Supplementary Materials

The following are available online at https://www.mdpi.com/article/10.3390/nu14030405/s1, Table S1: Changes in homeostasis glucose parameters after 6 weeks of intervention, Table S2: Changes in systemic inflammatory, oxidative, and vasodilatory biomarkers after 6 weeks of intervention.
