# Supplementary material for: Effects of an Optimized Aged Garlic Extract on Cardiovascular Disease Risk Factors in Moderate Hypercholesterolemic Subjects: A Randomized, Crossover, Double-Blind, Sustainedand Controlled Study
Source: Nutrients. 2022 Jan 18;14(3):405. doi: 10.3390/nu14030405 (PMC8838962; doi:10.3390/nu14030405)
Supplement: Supplementary file 1 [file nutrients-14-00405-s001.zip › nutrients-1508694-supplementary.pdf]

Supplementary Table S1. Changes in homeostasis glucose parameters after 6 weeks of intervention.

	Intervention				Change inter-treatments	
**Variable**	**AGB**		**Placebo**		**AGB *versus* Placebo**	
	**Baseline**	**Change**	**Baseline**	**Change**	**Mean (95%CI)**	**P-valor**
Glucose, *mg/dL*	94 ± 9.1	0.869 (-0.49; 2.2)	94 ± 10.0	0.849 (-1.0; 2.7)	-0.05 (-2.3;2.3)	0.997
Insulin, *μU/mL*	0.72 ± 0.24	-0.020 (-0.06; 0.02)	0.70 ± 0.26	-0.015 (-0.05; 0.02)	-0.005 (-0.06;0.05)	0.875
HOMA-IR (log)	0.08 ± 0.26	-0.015 (-0.06; 0.03)	0.06 ± 0.29	-0.011 (-0.05; 0.03)	-0.004 (-0.07;0.06)	0.899
QUICKI	0.37 ± 0.04	0.002 (-0.004; 0.008)	0.38 ± 0.04	0.001 (-0.005; 0.007)	0.001 (-0.008;0.01)	0.783

Abbreviations: AGB, Aged Black Garlic; Change, change from baseline. HOMA index for insulin resistance was calculated as: (insulin x glucose)/405. QUICKI index for insulin sensitivity was calculated as: 1/(log insulin + log glucose). Data expressed as mean ± standard deviation or mean (95% Confidence Interval, CI). ANCOVA Model adjusted by sex, age, and sequence of SANEs administration.

Supplementary Table S2. Changes systemic inflammatory, oxidative, and vasodilatory biomarkers and after 6 weeks of intervention.

	**Intervention**				**Change inter-treatments**	
**Variable**	**ABG**		**Placebo**		**ABG *versus* Placebo**	
	**Baseline**	**Change**	**Baseline**	**Change**	**Mean (95%CI)**	**P-valor**
hs-CRP (log), *mg/dL*	-0.75 ± 0.41	-0.002<br>(-0.08; 0.07)	-0.78 ± 0.41	0.029<br>(-0.04; 0.10)	-0.031<br>(-0.07; 0.14)	0.561
IL-6 (log), *pg/mL*	0.20 ± 0.25	0.002<br>(-0.07; 0.07)	0.20 ± 0.26	0.028<br>(-0.04; 0.10)	-0.025<br>(-0.07; 0.12)	0.613
MCP-1	1.90 ± 0.21	-0.046<br>(-0.09;-0.003)	1.91 ± 0.19	-0.073<br>(-0.11;-0.03)	0.030<br>(-0.03; 0.09)	0.364
LDLox, *U/L*	121 ± 34	-37.4<br>(-51; -24)	116 ± 30	-36.2<br>(-50; -22)	-1.21<br>(-21; 18)	0.902
NO (log), *µM*	1.38 ± 0.22	0.037<br>(-0.03;0.11)	1.41 ± 0.22	0.023<br>(-0.05;0.09)	0.014<br>(-0.11;0.09)	0.780

Abbreviations: ABG, Aged Black Garlic; Change, change from baseline; hs-CRP, high-sensitivity C reactive protein; IL-6, interleukin-6; MCP-1, monocyte chemotactic protein-1; LDLox, oxidized low density lipoproteins; NO, nitric oxide. Data expressed as mean ± standard deviation or mean (95% Confidence Interval, CI). ANCOVA Model adjusted by sex, age, sequence of treatments administration.
